# Applications of Micro/Nanoparticles in Microfluidic Sensors: A Review

**DOI:** 10.3390/s140406952

**Published:** 2014-04-21

**Authors:** Yusheng Jiang, Hui Wang, Shunbo Li, Weijia Wen

**Affiliations:** 1 College of Communication Engineering, Chongqing University, Chongqing 400044, China; E-Mail: 20121202036@cqu.edu.cn; 2 Department of Physics, Hong Kong University of Science and Technology, Clear Water Bay, Kowloon, Hong Kong; E-Mails: sliab@ust.hk (S.L.); phwen@ust.hk (W.W.)

**Keywords:** micro/nanoparticle, PDMS, microfluidic chip, electric property, magnetic property

## Abstract

This paper reviews the applications of micro/nanoparticles in microfluidics device fabrication and analytical processing. In general, researchers have focused on two properties of particles—electric behavior and magnetic behavior. The applications of micro/nanoparticles could be summarized on the chip fabrication level and on the processing level. In the fabrication of microfluidic chips (chip fabrication level), particles are good additives in polydimethylsiloxane (PDMS) to prepare conductive or magnetic composites which have wide applications in sensors, valves and actuators. On the other hand, particles could be manipulated according to their electric and magnetic properties under external electric and magnetic fields when they are travelling in microchannels (processing level). Researchers have made a great progress in preparing modified PDMS and investigating the behaviors of particles in microchannels. This article attempts to present a discussion on the basis of particles applications in microfluidics.

## 1. Introduction

Lab-on-a-chip (LOC) refers to a system that integrates one or several laboratory functions on a single chip of only millimeters to a few square centimeters in size. LOC systems have a promising future in simplifying sensing schemes and reducing parallel equipment needs, by implanting internal detection and processing modules in one signal chip. Microfluidics and nanofluidics, which deals with the behaviors, precise control, and manipulation of fluids that are geometrically constrained to a small, typically sub-millimeter scale, is considered as an appropriate approach for a small sensing system. There were several typical microfluidic components in this system [1], including microfluidic mixers, microheaters, micropumps, microdroplet controllers, and reaction chambers, which are fabricated from PDMS-based composites. In microfluidic systems, people always have to deal with particles either in the fabrication process or in the analyzing applications [2,3], for example, constructing conducting or magnetically flexible chips [4], GERF (giant electrorheological fluid) in microfluidics [5], micro/nano-particles, even biological particles (cells, DNA and proteins) [6]. The behaviors of particles in microfluidics become critical in research. Usually, the electric and magnetic properties should be considered. The common material polydimethylsiloxane (PDMS) [7] is a silicon-based organic polymer that has been widely used in the microfluidic chip fabrication owing to its good elastic properties, non-toxicity, biocompatibility, optical transparence, nonflammability, chemical inertness, as well as conformability, among other attributes. The combination of nanoparticles and PDMS provides flexibility in fabricating microfluidic devices for different applications.

In this paper, we review the applications of particles in microfluidic systems to illustrate how to alter the electric and magnetic property of PDMS by incorporating particles and how to manipulate particles on chips according to their electric and magnetic responses.

## 2. Electric Behaviors of Particles and Their Applications in Microfluidics

### 2.1. Design and Fabrication of PDMS-Based Conducting Composites

**Figure 1.**
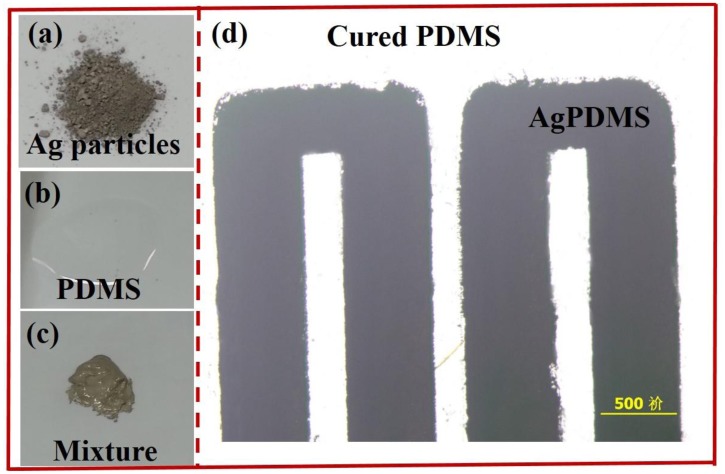
(**a**) The silver submicron particles; (**b**) PDMS pre-polymer; (**c**) The mixture of silver particles and PDMS gel; (**d**) Picture of conductive circuit with PDMS-based conducting composite (AgPDMS) on a pure PDMS slab (transparent part).

PDMS is a non-conducting material, on which patterning metallic structures during fabrication is challenging due to the weak adhesion between metals and PDMS. Therefore, the integration of conducting components in PDMS-based device has been a critical issue, especially for those applications related to electric manipulation and detection, e.g., electroosmotic pumps, DEP (dielectrophoresis) devices, biosensors, electro-rheological (ER) actuators and microheaters *etc.* Recently, Niu *et al.* [8] developed a method of patterning conductive structures using PDMS-based conducting composites, synthesized by uniformly mixing conductive particles, including silver particles and nanometer sized carbon particles, with PDMS gel. The silver and carbon black particles were easy to mix with the PDMS gel due to their desirable wetting characteristics. The conductivities of the two types of composites increased rapidly when the concentrations exceeded threshold values of 83 and 10 wt%, respectively. In the experiment, they chose about 1–2 μm silver particles and 10–100 nm carbon black particles as the additives to PDMS, and the gel was embedded into a photoresist mold on a glass substrate to pattern the conductive composites. Silver particles and their mixture with PDMS gel are shown in Figure 1a,c. Optical microscopy image of an electric circuit fabricated with conducting PDMS composites is shown in Figure 1d.

PDMS-based conducting composites exhibit good electrical conductivity and mechanical reliability [1]. By using this type of composites planar and 3D conducting microstructures can be constructed by the way of soft lithography. The breakthrough of this method was the development of a technique to incorporate soft conducting structures for polymer-based microfluidic chips since this composite is compatible with PDMS and it can use all these existing technologies for polymer patterning. Many other researchers also reported their works about the fabrication of conductive PDMS composites and their applications in different fields. Brun *et al.* [9] applied nanocomposite carbon/PDMS material with a ratio of 25% for chip-based electrochemical detection. In 2010, Scott *et al.* [10] synthesized gold nanoparticle/PDMS nanocomposites in different forms, such as foams, gels and films, all of which have distinctive structure and morphology. They demonstrated the capability of this composite in water purification and drug delivery systems for their chemically selective uptake and in releasing a fluorescent dye. Then, Niklaus and Shea [11] developed another method (ion implantation) to mix metal particles with PDMS. The volume fraction of metal was accurately controlled to form a composite layer less than 30 nm. Stassi and Canavese [12] prepared nickel/PDMS composites from spiky-shaped nickel powder with diameter in the range of 3.5–7 μm. They demonstrated a tunable electrical conductivity of up to nine orders of magnitude. All these kinds of conducting composites have applications in electric sensors, electrochemical reactions, electrokinetics of fluid, on-chip manipulation of particles and droplets, *etc*.

### 2.2. GERF Application in Microfluidics

Giant electrorheological fluid (GERF) [13,14] is a kind of smart material which has reversible characteristics of liquid solid transition under external electric field. This material actually shows the particles' electric behaviors. The main part of this fluid is barium titanyl oxalate nanoparticles. The suspending medium is silicon oil. These particles have very strong dipole-dipole interactions when an external electric field is applied. Then, they will form chain structures and solidify. In microfluidics, the size is very small compared to common equipment, indicating that the applied voltage could be reduced to achieve certain electric field strength (that is 500 V in microfluidic chips compared to 50,000 V in common equipment). The application of GERF in microfluidics is very straightforward—GERF based actuators [15], valves [16] and micromixers [17,18].

One simple example of microfluidic application of GERF is for realizing a microvalve inside a microfluidic chip. The chip was mainly composed of two layer structures. The lower layer was the control layer, which determines the status of the main reaction channel located in the upper layer. Elastic PDMS diaphragms sandwiched between the lower and upper layers serve to separate the fluids in the two layers. Two pairs of electrodes embedded on the sides of lower microfluidic channels serve as signal input. When an electric signal is applied, the GERF will solidify and the pressure inside the lower channel will increase dramatically. Then the PDMS diaphragms will deflect and block the fluid flow in the upper channel. This is defined as the off-state. When no signal is imposed, the fluid flows normally, and this is the on-state.

**Figure 2.**
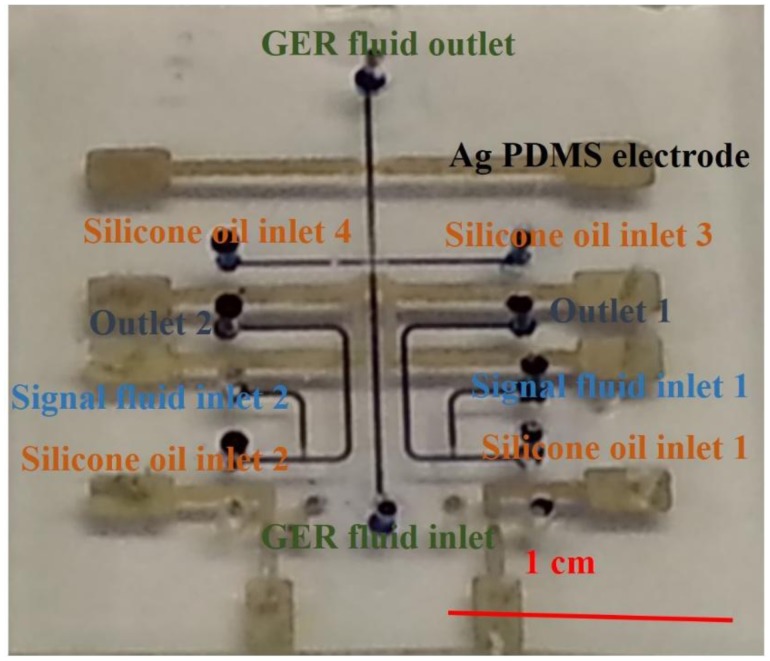
Optical image of the universal logic gate chip based on GER fluid.

Another application of GERF is microfluidic logic control [19], which is a new finding and has a promising future in simplifying control schemes and reducing parallel equipment needs by implanting internal droplet signal detection and processing modules inside microfluidic chips. Recently, researchers have successfully realized 16 kinds of microfluidic chip-based logic functions [20]. The chips are identical logic control components. The integrated chip was fabricated by soft lithography using PDMS-based conducting composites with embedded Ag/carbon-PDMS electrodes. Figure 2 shows a microfluidic logic gate based on GER fluid. All the channels are connected by conducting structures to transfer the signal. When GERF is present between two electrodes in the microchannel, the electrodes form a complete circuit and the voltage share is known. On the other hand, the circuit is an open circuit when no GERF flows between these paired electrodes. Based on this principle, one can adjust the design of microstructure as well as the voltage applied on the electrodes to achieve a certain logic function.

### 2.3. Particles' Electric Force in Microfluidic Channels

There are two main forces acting on the particles when an electric field is applied, namely, electrophoretic force and dielectrophoretic force. Usually, the particles are dispersed in aqueous solutions with low concentrations and the viscosity is almost the same as pure water compared to highly viscous GERF whose fluid medium is oil. The electrophoretic force could be expressed as [21]:

(1) $${\vec{u}}_{EP}=\mu_{EP}\vec{E}$$

where *μ_EP_* = − *ε_m_ζ_p_* / *η* is electrophoretic mobility. ε_m_ is the permittivity of the suspending medium, while η is the dynamic viscosity of the suspending medium. ζ_p_ represents the zeta potential of the particle. E is the electric field vector. Equation (1) shows that the EP (electrophoresis) velocity is linearly proportional to the local electric field. The direction of electrophoretic force is along the electric field lines, meaning the particles would travel along the channel when electric signal was imposed at the inlet and outlet.

If the electric field is non-uniform, the motion of the suspended particle is also affected by the dielectrophoretic force which is originated by the polarization of the particle in terms of an equivalent induced dipole moment. The time-average of this force, F_DEP_, on an insulating spherical particle is given by [21]:

(2) $${\vec{F}}_{\text{DEP}}=\left(1/2\right)\pi \varepsilon_{m}d^{3}f_{CM}\left(\vec{E}\cdot \nabla \vec{E}\right)$$

and:

(3) $${\vec{u}}_{\text{DEP}}=\mu_{\text{DEP}}\left(\vec{E}\cdot \nabla \vec{E}\right)=\left(\varepsilon_{m}d^{2}f_{CM}/6\eta \right)\cdot \left(\vec{E}\cdot \nabla \vec{E}\right)$$

where d is the diameter of particle, *f_CM_* = (*σ_p_* − *σ_m_*)/(*σ_p_* + 2*σ_m_*) is known as the Clausius-Mossotti (CM) factor, *σ_p_* and *σ_m_* are the electric conductivities of particle and the suspending medium, respectively. If the particle is less conductive than the suspending medium (*σ_p_* < *σ_m_*), then the CM factor will be negative (*f_CM_* < 0), resulting in a negative DEP force, which repels the particle away from the strong electric field region. Usually, the DEP force of a particle is exerted along the width of the microchannel (electrodes on the sidewall) or transversally across the height of the microchannel (electrodes on the top and bottom surface) to redirect particles in microfluidics.

**Figure 3.**
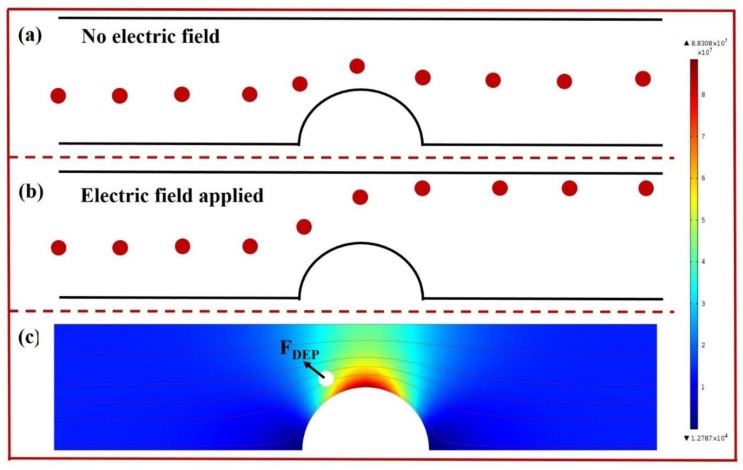
(**a**) Illustration of particles' trajectory when they travel through a channel neck; (**b**) Comparison of particles' trajectory when they travel through a channel neck and electric field applied; (**c**) Numerically simulated electric field distribution (E^2^) around the channel neck. Particles will experience large DEP force at the channel neck.

By applying these two unique electric field behaviors, particle manipulations including focusing, sorting, and enrichment have been demonstrated in a single microfluidic chip. In these systems, both DC and AC electric fields could be used to generate the forces acting on particles [22,23]. Moreover, the microchannel structure provides us another way to manipulate particles and biological cells [24]. Figure 3 illustrates the manipulation of particles in microchannels based on electrophoresis and dielectrophoresis when a DC signal is applied. The electric field gradient is generated by the structures inside the microchannel.

The applications of particles' electric force are mainly concentrated in the biomedical and biotechnological sciences. Many types of bioparticles have been investigated in this study, including blood cells, stem cells, neurons, pancreatic β-cells, bacteria and yeast, cell viability, apoptosis, viruses, even DNA and proteins [25].

## 3. Magnetic Behaviors of Particles and Their Applications in Microfluidics

### 3.1. Design and Fabrication of Magnetic PDMS Composites

Usually, it is important to employ various kinds of magnetic materials to achieve multiple functions in microfluidic chips. However, most materials used in fabricating microfluidic chips lack magnetic properties. Therefore, the processes aimed at transforming pure PDMS into a magnetic composite by the addition of magnetic nanoparticles are a critical issue in microfluidics, for instance, using carbonyl iron-PDMS (CI-PDMS) composite membranes and magnetorheological elastomers (MREs) [26].

The fabrication processes of CI-PDMS composites include the preparation of PDMS mixtures and addition of highly saturated magnetization CI powder. Detailed information can be found in [4]. The carbonyl iron particles are commercially available for purchase.

**Figure 4.**
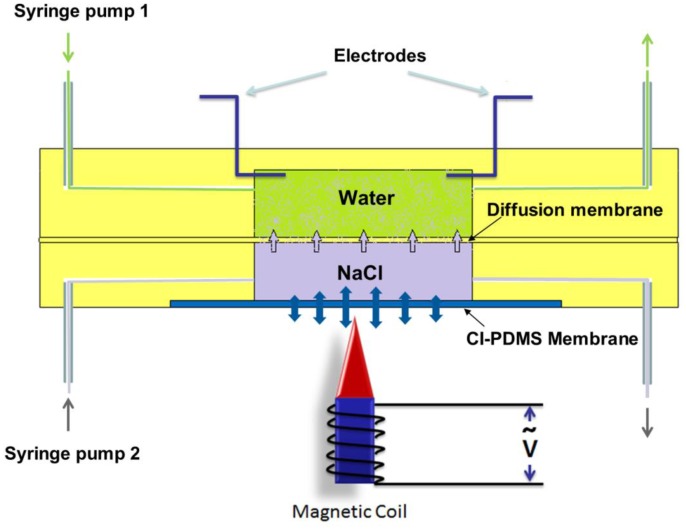
Cross-sectional view of a microfluidic device for chemical release. Reproduced with permission from [32].

In the resultant CI-PDMS composites, the CI particles on the surface were not directly exposed to the air reducing the risk of particle oxidation. Weight ratios of CI to PDMS ranging from 0.5 to 4.0 were tested in experiments to find the best one for application. The optimized membrane CI/PDMS ratio is around 2, which could be used to design and fabricate the largest deflection magnetic membrane [4]. The advantages of CI-PDMS composite membranes include easy fabrication, compatibility with PDMS, high magnetization and large deflection. One can use this material in microfluidic devices, especially for active fluid control by imposing a relatively low magnetic field. Figure 4 shows the application of CI-PDMS composites in controlled chemical release in microfluidics. The actuation of the magnetic membrane will alter the diffusion rate from the lower chamber to upper chamber. They also demonstrated the controlled drug release in culturing *Escherichia coli*. One can also prepare anisotropic CI-PDMS membrane by applying magnetic fields during the curing of PDMS [27]. This type of nanoparticle-based magnetic composites have wide applications in actuators [28,29] and micropumps [30,31].

**Figure 5.**
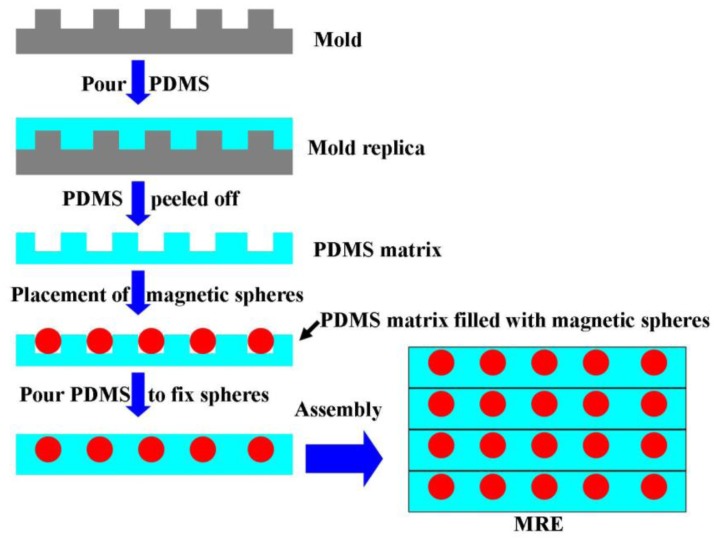
A typical fabrication process for MRE.

MRE [33] is another type of magnetic composite where highly elastic polymer matrices are filled with magnetic particles and operated within the pre-yield regime. In the fabrication process of patterned MREs [26], PDMS was chosen as a matrix and pure iron balls were used as dispersed particles. Figure 5 illustrates a typical fabrication process for MRE. A patterned mold which was a methyl-methacrylate board with regular holes etched by laser was prepared first. PDMS was then poured onto this mold to transfer the pattern. After that, pure iron balls were filled into the PDMS holes, followed by pouring a thin layer of PDMS on the front surface of the iron balls, so as to fix the position of the particles and clear the voids. After curing of the PDMS, one layer of MRE embedded with patterned magnetic iron particles was prepared. Then several layers with designed position and thickness were bond together, and the gap was filled with PDMS. Finally the patterned MRE were cured at a constant temperature in a vacuum oven. By using different molds and overlapping positions, different MRE structures can be obtained, such as lattice structures or body centered cubic (BCC) structures. The MRE samples with both of these two structures have field-dependent mechanical properties.

In 2013, a novel MRE design which consisted of multilayer thin MRE sheets bonded onto multilayer thin steel plates was reported [34]. This design could alter the lateral stiffness and damping force in real time and it has application in structural control in civil engineering. MRE are widely used in structures, devices, and instruments to reduce vibrations and noise [35,36].

### 3.2. Particles' Magnetic Force in Microfluidic Channels

Magnetic nanoparticles and microparticles which can be magnetically manipulated using permanent magnets or electromagnets have demonstrated new microfluidics applications in magnetic separation, immunoassays, drug delivery and hyperthermia studies [37]. The magnetic force of particles is obtained using an effective dipole moment approach expressed by [38]:

(4) $${\vec{F}}_{m}=\mu_{f}\left({\vec{m}}_{p,\text{eff}}\cdot \nabla \right){\vec{H}}_{a}$$

where *μ_f_* is the permeability of the transport fluid, *m⃑_p,eff_* is the effective dipole moment, and *H⃑_a_* is the external magnetic field intensity. Therefore, the magnetic particles could be manipulated by the external magnetic fields.

**Figure 6.**
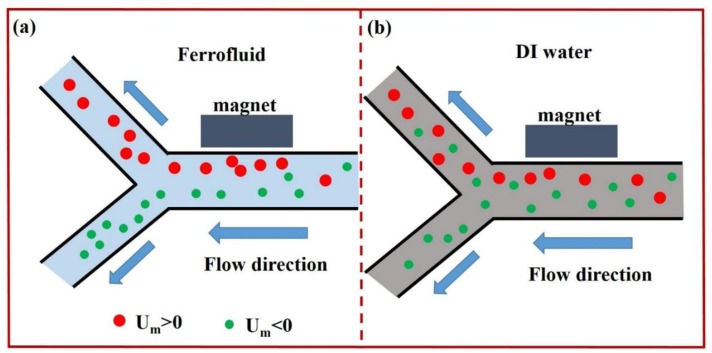
Schematics illustrating and comparing the separation mechanisms of magnetic and diamagnetic particles in ferrofluid (**a**) and DI water (**b**). U_m_ > 0 and U_m_ < 0 indicate the positive and negative magnetophoresis experienced by the magnetic and diamagnetic particles, respectively.

On-chip magnetic particle manipulation is an active research field these years. In 2005, Mirowski *et al.* [39] realized on-chip particle manipulation by an external magnetic force microscope according to the particle's magnetic property. The precision of this design could reach down to nanometer. In 2007, they performed magnetic particle transport and sorting by an array of magnetic spin valves with bistable ferromagnetic and antiferromagnetic net magnetization states [40]. In 2013, Liang *et al.* [41] demonstrated separation of particles based on the magnetic field induced effect. They enhanced the separation efficiency by replacing traditional DI water by ferrofluid which has negative magnetophoresis compared to suspending particle's positive magnetophoresis. A schematic diagram of this separation based on magnetic force is shown in Figure 6.

Giant magnetoresistance (GMR) is another useful technique for on-chip manipulation and detection of magnetic particles [42,43]. Microdroplets containing magnetic particles in silicon oil can be displaced, merged, mixed and separated on a magnetic platform [44]. Compared to electric fields which may destroy the viability of biological cells, magnetic fields have less negative impact on cells. Usually, magnetic particles could be modified by chemical methods to bind living cells and thus the living cell separation is conducted by a magnetic field generated either on chip or off chip. One example was demonstrated by Xia *et al.* [45] to separate *E. coli* and human red blood cells (RBCs). Other application of magnetic particle for bioresearch such as biosensing [46,47], microfluidic cytometry [48], rare cell separation [49] and pathogen detection [50] was also achieved by applying magnetic force and motion. This technique is regarded as simple, fast, inexpensive and easy to operate.

## 4. Conclusions

Nanoparticles such as carbonyl-iron, carbon black and silver particles can be mixed with PDMS to achieve magnetic or conductive composites. This type of composites enabled a methodology for constructing planar and 3D microstructures via soft lithography. Different patterns of electrodes can also be fabricated by soft lithography using these materials, and GERFs can be incorporated into electrodynamic devices to realize controllable sample mixing, microvalves and micropumps, *etc*. The electric and magnetic particles' behaviors in microfluidic channel is another useful technique in LOC sensing systems. The electric force is mainly contributed by electrophoresis and dielectrophoresis, both of which could be utilized to focus, trap, and sort particles. The magnetic force refers to dipole force and manetoresistance in the external magnetic field generated either by on-chip or off-chip electromagnets. Both of electric and magnetic behaviors in microchannel could be applied in biological sensing and processing, such as drug delivery, cell separation, detection, immunoassay, *etc*.
